# Clinical Outcomes and Complication Rates of Ventriculoperitoneal Shunts in Hydrocephalic Infants with Meningomyelocele: A Ten-Year Review at a Single Institution

**DOI:** 10.3390/children11121508

**Published:** 2024-12-11

**Authors:** Çağlar Türk, Umut Tan Sevgi, Eda Karadağ Öncel, Mahmut Çamlar, Ozan Akgül, Füsun Özer

**Affiliations:** 1 Department of Neurosurgery, Health Sciences University, Izmir City Hospital, 35540 Izmir, Turkey; caglar.turk@saglik.gov.tr (Ç.T.); mahmut.camlar@sbu.edu.tr (M.Ç.); ozan.akgul@sbu.edu.tr (O.A.);; 2 Department of Pediatric Infectious Diseases, Faculty of Medicine, Dokuz Eylül University, 35220 Izmir, Turkey; eda.karadagoncel@saglik.gov.tr

**Keywords:** external ventricular drainage, hydrocephalus, meningomyelocele, shunt infection

## Abstract

Background/Objectives: This study aimed to investigate the surgical treatment and management of hydrocephalus in infants with meningomyelocele and compare the single-center experience with the previous studies. Methods: This retrospective study included 81 infants (47 females and 34 males) who underwent meningomyelocele closure surgery and subsequent ventriculoperitoneal (VP) shunt surgery for hydrocephalus. Clinical and demographic data were retrospectively collected from hospital records, focusing on variables such as the timing of VP shunt placement relative to MMC closure, postoperative complications, and the need for shunt revisions. Patients were followed for a mean duration of 58.11 months to monitor long-term outcomes and identify factors associated with shunt failures and infections. Results: The mean follow-up period since birth was 58.11 (33.72) months. Shunt problems affected 30% (25/81) of patients with mechanical causes (8/25) and infections (6/25). A proximal mechanical malfunction/dysfunction was seen in 32% (8/25) of the shunts. Shunt infections occurred in 23% (19/81) of infants, and the mean time for shunt infection onset following the VP shunt procedure was 0 (0–39) median (min–max) months. Overall, 8 (9.9%) infants had short-term shunt infections, whereas 11 (13.6%) had long-term shunt infections. The mean length of the intensive care unit stay was 35.75 (25.28) days. Significant difference was seen in the number of shunt reoperations for short- and long-term infections (*p* < 0.001). All infants had at least one operation before the infection of their shunt system. Male gender was significantly associated with long-term shunt infections (*p* = 0.021). The study revealed methicillin-resistant coagulase-negative staphylococcus to be the most common isolated organism from infected shunts at 72.7% (6/11). Conclusions: This study demonstrates that hydrocephalic infants with meningomyelocele undergoing VP shunt surgery face notable risks of infection and mechanical complications, with methicillin-resistant coagulase-negative staphylococcus identified as the most common pathogen. The findings emphasize the importance of comprehensive postoperative care and targeted infection management to improve outcomes in this vulnerable population.

## 1. Introduction

Hydrocephalus is the presence of excess cerebrospinal fluid in the central nervous system, annually affecting around 400,000 new pediatric cases globally [1]. Around one in every four infants born with meningomyelocele (MMC) has hydrocephalus [2]. Moreover, hydrocephalus has an incidence and prevalence rate of 0.9–1.2 per thousand and 0.2–0.6 per thousand in developed countries, respectively [3]. While hydrocephalus is observed at birth in approximately 15–25% of infants with MMC [4,5], its incidence has been reported to increase to 57–86% following the postnatal closure of the MMC defect [5]. The reported high incidence of hydrocephalus after postnatal closure of the MMC defect underscores the need for timely diagnosis and effective management strategies to improve outcomes in this population.

Medical treatment for hydrocephalus management is far inferior to surgical treatment. Medical treatment may be used in surgically unsuitable patients to avoid shunt operations or delay the onset of neurological symptoms in those with shunts [6]. However, implantation of a ventriculoperitoneal (VP) shunt is the standard surgical treatment of hydrocephalus in MMC. This procedure transfers excess cerebrospinal fluid (CSF) from the brain’s ventricles to the abdominal cavity, where it is absorbed.

A VP shunt should be performed during surgical repair of myelocele to prevent CSF leakage via the spinal cord as it is a risk factor for shunt infection and should be avoided. Furthermore, a VP shunt promotes normal infant development by initially reducing intracranial hypertension. Persistent CSF leakage after an MMC operation indicates concurrent hydrocephalus [7].

The VP shunt procedure has several potential complications, including malfunction/dysfunction and infection. Two decades ago, McGirth et al. [8] reported 14% of shunt failures during the first month after installation. Recently, 8.8% of shunt failures were reported by Anderson et al. [9] in the first month of installation. About 80% of all shunts face mechanical complications, in which 33–50% occur within the first year after performing VP and 1–7% failure rate in the second year [10]. In addition, another cause of shunt complications are infections with 10% or lower report rate [10]. Infants under 6 months are more likely to develop infection complications, *Staphylococci* being the most common isolated microorganism.

This retrospective study aimed to investigate the clinical characteristics of infants with VP shunts implanted for hydrocephalus management who had previously undergone MMC closure operation, VPS infection management, and a comparison of early and late shunt infections.

## 2. Materials and Methods

### 2.1. Patients and Data Collection

This retrospective study included 81 infants (47 female and 34 male) who underwent meningomyelocele closure surgery and subsequent VP shunt surgery for hydrocephalus at the Department of Neurosurgery in our center between 1 January 2011 and 31 December 2021. Demographic and clinical information about the infants was obtained from patient files and center records. A case information sheet developed by the authors was filled for each infant with the following parameters: age, gender, time elapsed over MMC operation for VP shunt operation, preoperative head circumference, operation timing for hydrocephalus, the presence of surgical complication, status of VP shunt infection, preoperative CSF culture and its result, preoperative CSF cytology, status and site of shunt malfunction and dysfunction, status of CSF leakage in the operation wound, and admission to the emergency room due to revision/infection.

### 2.2. Diagnosis and Treatment Criteria and Categorization of Ventriculoperitoneal Shunt Infections

Shunt infection was confirmed in infants with at least one of the following criteria: the presence of an agent pathogen growth in CSF culture or nonculture-based microbiologic testing method (only when testing is performed for clinical diagnosis or treatment) or at least two of the following: fever (>38 °C) or headache (for patients ≤ 1 y of age, these clinical criteria can be met by fever [>38 °C], hypothermia [<36 °C], apnea, bradycardia, or irritability without other recognized cause); meningeal sign(s) with no other recognized cause; and cranial nerve sign(s) with no other recognized cause. Furthermore, at least one of the following was required: increased white cells, increased protein, and decreased glucose in CSF; organism(s) found in Gram CSF stain; organism(s) identified from the blood by culture or nonculture-based microbiologic testing method; and diagnostic single antibody titer (IgM) or fourfold increase in paired sera (IgG) for the organism [11].

Successful shunt infection treatment was proven in the absence of any infection symptom, lack of fever > 38 °C in the past 48 h, no agent pathogen development in two consecutive sterile CSF cultures, CSF glucose > 20 mg/dL, CSF protein < 200 mg/dL, and negative Gram CSF stain [12,13,14].

This study divided shunt infections into two types: short-term (early) and long-term (late), based on the period between shunt installation and the development of infection. Shunt infections during the first year after the installation of the VP shunt are described as “short-term shunt infections”, whereas those after the first year are described as “late-term shunt infections” [15].

Infants with shunt infections during follow-up were hospitalized for external ventricular drainage after the shunt was removed due to infection. The infants that had surgery were given appropriate antibiotic medication. When CSF cultures revealed no infection, they had reoperation. Almost all infants with shunt dysfunction had their shunts revised after being admitted to the emergency department.

Short- and long-term shunt infections were evaluated based on their follow-up period from birth, gender, preoperative head circumference, comorbidity, complications, median CSF glucose and protein levels, mechanical shunt dysfunction, number of shunt reoperations, day of VP operation after MMC operation, time of VP shunt operation after MMC operation, length of intensive care unit (ICU) stay, and mortality.

### 2.3. Statistical Analysis

The variables were analyzed using SPSS 25.0 (IBM Corporation, Armonk, New York, NY, USA). The Shapiro–Wilk–Francia test was performed to determine if the data conformed to the normal distribution, and the Levene test was used to assess the variance homogeneity. Using Monte Carlo findings, the Mann–Whitney *U* test compared two independent groups based on quantitative variables. Fisher’s exact test with Monte Carlo simulation was used to compare categorical variables. Quantitative variables were expressed as mean (standard deviation) and median (minimum–maximum) in the tables, whereas categorical variables were expressed as *n* (%). Variables were examined at a 95% confidence level, with a *p*-value of 0.05 indicating statistically significant.

The design and protocol were approved by the Institutional Ethics Committee (Number: 2022/12-16), and this study is in accordance with the principles of the Helsinki Declaration.

## 3. Results

This retrospective analysis encompassed infants who underwent VP shunt installations following MMC closure, all of which were conducted at our medical facility. Overall, 22 infants had comorbidities such as inguinal hernia, neurogenic bladder, colon perforation, tethered cord, kyphoscoliosis, clubfoot, and developing hip dysplasia that required repair surgery. The mean follow-up period for infants since birth was 58.11 (33.72) months.

The sociodemographic data and clinical characteristics of the infants are presented in Table 1. The mean preoperative head circumference, mean preoperative CSF glucose level, and mean CSF protein level of the infants were 36.74 (9.87) cm, 36.64 (9.87) mg/dL, and 67.06 (27.63) mg/dL, respectively. No perioperative complications and pathogenic agent were identified in perioperative CSF cultures. In total, 36 (44.4%) infants had a VP shunt procedure 1–7 days after the MMC operation, whereas 45 (55.6%) had surgery > 7 days later. The mortality rate was 7.4%. The VP shunt procedure was performed on a mean of 11.02 (7.74) days after the MMC surgery. The mean number of shunt reoperations was median (min–max) 0 (0–3, Table 1).

Mechanical shunt dysfunction was observed in 19 (23.5%) infants. Table 2 shows the causes of shunt malfunction in the infants in our study group. The proximal catheter was the most common source of mechanical dysfunction (*n* = 8). The VP shunt infection was the other cause of mechanical dysfunction (*n* = 6). In two infants, infection was associated with mechanical dysfunction in the shunt valve, and in three infants, infection was associated with mechanical dysfunction in the proximal region of the shunt.

Methicillin-resistant coagulase-negative staphylococcus (MRCoNS) was the most isolated pathogenic agent identified from the shunts, infecting 8/11 (72.7%) infants. Gram-negative microorganisms were isolated from 3/11 (27.25%) infants: *Escherichia coli*, *Klebsiella pneumoniae*, and *Pseudomonas aeruginosa* (one infant each, Table 2).

The mean time of onset of postoperative shunt infection from the VP shunt placement was 0 (0–39) months. Short-term shunt infection occurred in eight (9.9%) of the hydrocephalic infants, and long-term shunt infection occurred in only 11 (13.6%) infants. The mean length of stay in the infant ICU was 35.75 (25.28) days (Table 1). Because all eight infants with short-term infection also developed a long-term infection, dividing the groups and presenting the statistical analysis findings with a single *p*-value made it statistically impossible to compare short-term and long-term shunt infections. Both categories had significantly higher rates of shunt reoperations (*p* < 0.001). Infants with short- and long-term infections underwent at least one operation before their shunt system was infected. In contrast, the male gender was statistically significant for the presence of long-term shunt infections (*p* = 0.021). The other variables had no statistically significant difference (Table 3).

## 4. Discussion

Meningomyelocele is the most common form of neural tube defect caused by improper spine closing before birth [16]. Several comorbidities are associated with this condition, hydrocephalus being the most common one [5]. Hydrocephalus in infants with myelomeningocele (MMC) develops due to multiple factors, mainly stemming from the anatomical and physiological abnormalities linked to the condition. Our study comprised 81 infants who suffered hydrocephalus due to MMC. All infants in this study underwent repair surgery for MMC and a VP shunt procedure at our center. Most infants with shunts will develop shunt dysfunction at some time, necessitating several revisions, including replacing a VP shunt. Shunt infection remains a significant cause of morbidity and mortality and increases disease burden of the country [17]. This study reports the management and surgical approach of hydrocephalic infants through a 5-y follow-up.

Comorbidities associated with MMC, such as urinary tract, intestinal, musculoskeletal, sensorineural problems, and scoliosis [18], increase the risk of shunt infections, particularly in infants with multiple conditions. Additionally, the need for various surgical treatments to address other congenital malformations further elevates this risk [19,20]. In our study, comorbidities including inguinal hernia, neurogenic bladder, colon perforation, tethered cord, kyphoscoliosis, clubfoot, and developmental hip dysplasia were seen in 22 infants. The infants required repair surgeries for these anomalies during their follow-up period. In this study, about 27% (22/81) of the infants were found to have shunt infections, which represented the most prevalent cause of complications. Additionally, 23% (19/81) presented with multiple congenital anomalies.

Particularly in the short term, shunt infections and malfunction/dysfunction are the leading causes of shunt failure and neurosurgeons must take all necessary precautions [21,22]. In present study, 30% (25/81) of infants had shunt complications throughout their 5-y follow-up, including mechanical causes (8/25) and infections (6/25), consistent with the literature [11].

After the VP shunt is performed, ventricular catheter obstruction due to debris, coagulum, or the choroidal plexus is a common cause of shunt malfunction/dysfunction. Proximal ventricular end obstruction is frequently encountered in children due to contact with a small brain or ventricular size. It can be observed in the early postoperative period [23]. Although proximal ventricular catheter obstruction is a common cause of shunt malfunction, distal complications also play a significant role in the postoperative period. Piatt et al. argued that distal malfunction was more prevalent than proximal malfunction/dysfunction. Moreover, they noted that distal malfunction tends to occur later in the postoperative period, making it time-dependent [14]. In our study, 32% (8/25) of the patients experienced isolated proximal mechanical malfunctions/dysfunctions without concurrent infections, valve issues, or distal complications. Only one patient showed similar isolated issues in the distal region. This pattern, where proximal issues predominate, aligns with the majority of the literature [23,24].

Infection is the second leading cause of shunt malfunction/dysfunction, with a reported rate of approximately 8–15% among patients who underwent VPS placement [16,25]. Previous studies on shunt infections have found that infants with MMC had higher shunt infection rates than pediatric patients with hydrocephalus [11,26]. The shunt infection rate for hydrocephalic infants due to MMC has been reported to be 13.6–23% in the literature [2,27]. The shunt infection rate in our study was 13.5%, which positions it near the lower boundary of the rates reported in the literature.

The most common causative agents for VP shunt infections in hydrocephalic infants reported in the literature are Gram-positive, such as *Staphylococcus epidermidis* (52.8–88.9%), *Staphylococcus aureus* (12–40%), *Streptococcus Group B*, and *Enterococcus* species. However, Gram-negative bacilli, including Enterobacter species, K. pneumoniae, and P. aeruginosa, are also identified in 9–22% of cases [28,29,30]. MRCoNS is becoming more common worldwide with antibiotic-resistant complex cases [31]. In our study, MRCoNS 72.7% (8/11) was the most commonly isolated organism from infected shunts. Gram-negative microorganisms were isolated from three infants: *E. coli*, *K. pneumoniae*, and *P. aeruginosa* (one infant each). In this context, our results are consistent with the literature.

When VP shunt infections are classified based on the time from shunt installation to infection onset, infections within the first year are classified as “short-term shunt infections”, whereas those after the first year are classified as “late-onset shunt infections” [15]. In our study, the onset of shunt infection occurred 0 months after surgery. In the literature, VP shunt infections were reported in the first 3 months, with younger infants experiencing the infection sooner [31]. Short-term shunt infection occurred in eight (9.9%) of the hydrocephalic infants, and long-term shunt infection occurred in only 11 (13.6%) infants.

Infants with short-term and long-term shunt infections had significantly higher shunt reoperation rates (*p* < 0.001). The pathogenesis of shunt infections is caused by distal end-related retrograde infections, perioperative colonization, hematogenous contamination, and CSF leakage from the surgical site [32]. In our study, approximately one-third of the shunts had a mechanical malfunction in the proximal region. Proximal shunt infections are less common than distal ones; however, they are often associated with perioperative inoculation of infectious agents, which can result in local inflammation around the scalp incision site [33].

Infants with short- and long-term infections underwent at least one operation before their shunt system was infected. Newborns in this study were more likely to develop infection as the number of surgeries increased [32,34]. There was no significant difference in ICU stay duration and mortality rates between short- and long-term shunt infections.

However, male gender was statistically significant in the presence of long-term shunt infections (*p* = 0.021). A previous study revealed that male gender was associated with an increased risk of shunt complications [35]. Several studies have shown that males are more likely to develop shunt infections than females. The reasons for this gender difference remain unclear; however, several hypotheses have been proposed [19,36,37,38]. Anatomical differences might be one possible explanation. Males might be susceptible to infection during the surgical procedure due to the proximity of the shunt tubing with the genital area as the urethra closer to the shunt tubing may be a source of bacterial colonization and subsequent infection [39].

Hormonal differences between males and females have also been identified as contributing factors. Hormones play an essential role in immune system regulation and hormonal variations in males might make them more susceptible to shunt infections [40].

Additionally, behavioral differences between males and females may contribute to the increased risk in males. Males are more active and participate in rough play, which can cause trauma or damage to the shunt system, serving as an entry point for bacteria and increasing the probability of infection [41].

Hydrocephalus mortality rates in infants with MMC ranged from 1 to 48% [42,43,44]. Recent advancements in shunt design, surgical procedures, and infection prevention resulted in comorbidity-related deaths ranging from 9 to 43%. Shunt infections account for <one-third of deaths [42,45,46]. The total mortality reported in present study was 7.4%, which is consistent with the previous literature.

Although performing statistical analysis to determine trends over the years is challenging due to the heterogeneity of patients—with multiple comorbidities—and the context of low- and middle-income countries, over the ten-year duration of our study, we have observed a perceived improvement in surgical outcomes for hydrocephalic infants with meningomyelocele at our institution. This improvement is likely attributed to the implementation of a multidisciplinary approach and the accumulation of surgical experience over the years. Close collaboration among neurosurgeons, pediatricians, infectious disease specialists, urologists, orthopedic surgeons, and rehabilitation therapists has enabled comprehensive management of the complex comorbidities these patients often face—such as urinary tract anomalies, neurogenic bladder, bowel dysfunction, musculoskeletal deformities, and other congenital anomalies—that can increase the risk of infections compromising the shunt system. By proactively addressing these issues through early diagnosis, regular monitoring, and appropriate interventions, we aim to minimize their impact on neurosurgical outcomes. Advancements in perioperative care—including stricter infection control protocols, meticulous surgical techniques, and improved postoperative monitoring—have also contributed to better patient outcomes. Our experience underscores the importance of a holistic, multidisciplinary approach in treating this vulnerable population, emphasizing that continued collaboration among specialties and the ongoing enhancement of surgical expertise are essential to further reduce complication rates.

## 5. Limitations

This single-center study had some limitations, including a retrospective design that limits the power of statistical analysis, lack of a predefined surgical guidelines, direct impact on study results for follow-up, and determining the risk factor for recurrent infections.

## 6. Conclusions

Despite advancements in cerebrospinal fluid diversion techniques, endoscopic procedures, and improvements in shunt equipment for the central nervous system, treating hydrocephalus remains one of the most challenging issues in neurosurgery due to the increased risk of complications from invasive surgical interventions. This study demonstrates that hydrocephalic infants with meningomyelocele undergoing ventriculoperitoneal shunt surgery face notable risks of infection and mechanical complications, with methicillin-resistant coagulase-negative staphylococcus identified as the most common pathogen. These findings emphasize the importance of comprehensive postoperative care and targeted infection management to improve outcomes in this vulnerable population. Therefore, establishing universal management guidelines through multidisciplinary collaboration is essential to enhance patient care and reduce complication rates.

## 7. Future Directions

Advancements in shunt technology and surgical techniques offer promising avenues to reduce complication rates in hydrocephalic infants with meningomyelocele. The use of pressure-adjustable programmable shunts and antibiotic-impregnated shunt systems has shown potential in decreasing infections and mechanical failures [47,48], although more extensive studies are needed to confirm their long-term efficacy and safety. Additionally, fetal repair of meningomyelocele may reduce the severity of hydrocephalus and the subsequent need for shunt placement, warranting further investigation into its long-term outcomes [49]. Future research should focus on multicenter, prospective studies to evaluate these innovations, with collaborative efforts across specialties to develop standardized management protocols and improve patient outcomes in this vulnerable population.

## Figures and Tables

**Table 1:** Descriptive statistical data for hydrocephalic neonates with meningomyelocele.

Presence of Late Shunt Infection *	
No	70 (86.4)
Yes	11 (13.6)
Presence of Early Shunt Infection *	
No	73 (90.1)
Yes	8 (9.9)
Gender *	
Female	47 (58)
Male	34 (42)
Presence of Comorbidity *	
No	59 (72.8)
Yes	22 (27.2)
Perioperative Complication *	
No	81 (100)
Yes	0 (0)
Germ Growth in Perioperative CSF Culture *	
No	81 (100)
Yes	0 (0)
Mechanical Shunt Dysfunction *	
No	62 (76.5)
Yes	19 (23.5)
Time elapsed over MMC operation for VP shunt operation *	
1–7 Days	36 (44.4)
>7 Days	45 (55.6)
Mortality *	
Alive	75 (92.6)
Exitus	6 (7.4)
Patient Follow-up Periods from Birth (months) **	58.11 ± 33.72
Preop Head Circumference (cm) **	36.74 ± 3.9
Preop CSF Glucose **	36.64 ± 9.87
Preop CSF Protein **	67.06 ± 27.63
Number of Shunt Reoperations ***	0 (0–3)
On Which Day of VP Shunt Operation After MMC Operation **	11.02 ± 7.74
Time of Onset of Postop Shunt Infection (months) ***	0 (0–39)
Duration of ICU stay (day) **	35.75 ± 25.28

* *n*, % ** mean ± SD *** median (min–max); MMC; meningomyelocele, VP; ventriculoperitoneal, CSF; cerebrospinal fluid.

**Table 2:** Causes of malfunction and pathogens of the shunt infections of hydrocephalic neonates with meningomyelocele.

	*n* (%)
Cause of Malfunction	
Distal Obstruction **without Infection**	1 (4.0)
Distal Obstruction + **Infection**	0 (0.0)
**Only Infection**	6 (24.0)
Valve Dysfunction **without Infection**	1 (4.0)
Valve Dysfunction + Distal Obstruction	1 (4.0)
Valve Dysfunction + **Infection**	2 (8.0)
Valve Dysfunction + Proximal Obstruction	2 (8.0)
Proximal Obstruction **without Infection**	8 (32.0)
Proximal Obstruction + Distal Obstruction	1 (4.0)
Proximal Obstruction + **Infection**	3 (12.0)
**Shunt Infection** (**pathogens**)	
Methicillin-resistant coagulase negative staphylococcus	8 (72.7)
*Klebsiella pneumoniae*	1 (9.1)
*Escherichia coli*	1 (9.1)
*Pseudomonas aeruginosa*	1 (9.1)

**Table 3:** A comparison of infants with short-term and long-term shunt infections.

	Presence of Short-Term Shunt Infection			Presence of Long-Term Shunt Infection		
	No	Yes	*p*	No	Yes	*p*
	(*n* = 73)	(*n* = 8)		(*n* = 70)	(*n* = 11)	
(**Age**)**-Patient Follow-Up Periods from Birth, median** (**min–max**)	**52** (**2–116**)	40 (24–85)	0.475 ᵘ	51 (2–116)	52 (24–101)	0.887 ᵘ
**Gender** (**Male**)**, *n*** (**%**)	33 (45.2)	1 (12.5)	0.130 ᶠ	33 (47.1)	1 (9.1)	**0.021 ᶠ**
**Preop Head Circumference** (**Cm**)**, median** (**min**/**max**)	36 (30–56)	36.5 (34–40.5)	0.828 ᵘ	36 (30–56)	38 (34–43)	0.426 ᵘ
**Presence of Comorbidity, *n*** (**%**)	19 (26)	3 (37.5)	0.676 ᶠ	18 (25.7)	4 (36.4)	0.479 ᶠ
**Perop Complication, *n*** (**%**)	0 (0)	0 (0)	-	0 (0)	0 (0)	-
**Germ Growth in Perioperative CSF Culture, *n*** (**%**)	0 (0)	0 (0)	-	0 (0)	0 (0)	-
**Preop CSF Glucose, median** (**min**/**max**)	35 (17–66)	34.5 (24–51)	0.724 ᵘ	35.5 (17–66)	34 (24–51)	0.580 ᵘ
**Preop CSF Protein, median** (**min**/**max**)	64 (25–192)	58 (43–161)	0.847 ᵘ	64 (25–192)	55 (43–161)	0.968 ᵘ
**Mechanical Shunt Dysfunction, *n*** (**%**)	16 (21.9)	3 (37.5)	0.383 ᶠ	14 (20)	5 (45.5)	0.118 ᶠ
**Number of Shunt Reoperations, median** (**min**/**max**)	0 (0–3)	1 (1–3)	**<0.001 ᵘ**	0 (0–3)	1 (1–3)	**<0.001 ᵘ**
**On Which Day of VP Shunt Operation After MMC Operation, median** (**min**/**max**)	9 (1–38)	6.5 (3–31)	0.351 ᵘ	8.5 (1–38)	7 (3–31)	0.473 ᵘ
**Time elapsed over MMC operation for VP shunt operation** (**>7 Days**)**, *n*** (**%**)	42 (57.5)	3 (37.5)	0.456 ᶠ	40 (57.1)	5 (45.5)	0.526 ᶠ
**Duration of ICU Stay** (**Day**)**, median** (**min**/**max**)	26 (10–158)	51 (13–109)	0.069 ᵘ	25.5 (10–158)	48 (13–109)	0.121 ᵘ
**Mortality** (**Ex**)**, *n*** (**%**)	6 (8.2)	0 (0)	0.999 ᶠ	6 (8.6)	0 (0)	0.590 ᶠ

ᶠ Fisher Exact Test (Monte Carlo), ᵘ Mann–Whitney *U* Test (Monte Carlo), minimum, maximum, VP. ventriculoperitoneal, and MMC: meningomyelocele.

## Data Availability

The data presented in this study are not publicly available due to privacy and ethical restrictions. However, they can be made available from the corresponding author upon reasonable request and with permission from the respective ethics committee.
